# Predicting immune‐related adverse events using a simplified frailty score in cancer patients treated with checkpoint inhibitors: A retrospective cohort study

**DOI:** 10.1002/cam4.6013

**Published:** 2023-05-03

**Authors:** Cecilia Olsson Ladjevardi, Anthoula Koliadi, Viktoria Rydén, Ali Inan El‐Naggar, Evangelos Digkas, Antonios Valachis, Gustav J. Ullenhag

**Affiliations:** ^1^ Department of Immunology, Genetics, and Pathology Uppsala University Uppsala Sweden; ^2^ Department of Oncology Uppsala University Hospital Uppsala Sweden; ^3^ Department of Oncology, Faculty of Medicine and Health Örebro University Örebro Sweden; ^4^ Department of Oncology Mälarsjukhuset Eskilstuna Sweden

**Keywords:** advanced cancer, checkpoint inhibitor, cohort study, frailty score, immune‐related adverse events

## Abstract

**Objective:**

Checkpoint inhibitors (CPIs) are in widespread clinical use. Little is known about which patients are at risk for developing toxicity. It is essential being able to identify patients with higher risk of experiencing immune‐related adverse events (IRAEs) before initiation of CPI treatment to optimize treatment decisions and follow‐up strategy. The aim of this study was to investigate whether a simplified frailty score based on performance status (PS), age, and comorbidity expressed as Charlson comorbidity index (CCI) could predict development of IRAEs.

**Methods:**

We performed a retrospective cohort study at three Swedish centers. All patients (*n* = 596) treated with PD‐L1 or PD‐1 inhibitor for advanced cancer between January 2017 and December 2021 were included.

**Results:**

In total, 361 patients (60.6%) were classified as nonfrail and 235 (39.4%) as frail. The most common cancer type was non‐small cell lung cancer (*n* = 203; 34.1%) followed by malignant melanoma (*n* = 195; 32.7%). Any grade of IRAE occurred in 138 (58.7%) frail and in 155 (42.9%) non‐frail patients (OR: 1.58; 95% CI: 1.09–2.28). Age, CCI, and PS did not independently predict the occurrence of IRAEs. Multiple IRAEs occurred in 53 (22.6%) frail and in 45 (12.5%) nonfrail patients (OR: 1.62; 95% CI: 1.00–2.64).

**Discussion:**

In conclusion, the simplified frailty score predicted all grade IRAEs and multiple IRAEs in multivariate analyses whereas age, CCI, or PS did not separately predict development of IRAEs suggesting that this easy‐to‐use score may be of value in clinical decision making but a large prospective study is needed to assess its true value.

## 1 INTRODUCTION

Immunotherapy with checkpoint inhibitors (CPIs) has revolutionized the therapeutic strategies in various cancer types and in different treatment settings during the last decade.^1^, ^2^, ^3^ The number of cancer patients eligible for CPIs is expected to increase^4^ considering the rising number of ongoing randomized trials with CPIs, the increasing trend of new treatment indications for CPIs and not the least the growing cancer incidence.

In general, CPIs have a favorable toxicity profile compared to chemotherapy.^5^ However, patients treated with CPIs have a considerable risk of immune‐related adverse events (IRAEs) that may affect almost any organ system and may occur at any timepoint during treatment and even after treatment discontinuation.^6^ Although IRAEs are often reversible when management guidelines are followed accurately, some IRAEs are long‐term and life‐threatening.^7^, ^8^ Therefore, it is essential to be able to identify patients with higher risk for IRAEs in clinical practice before initiation of CPI treatment to optimize treatment decisions and follow‐up strategy.

Research in this field is ongoing and recent studies have focused on identifying predictive biomarkers for IRAEs through “‐omics” technologies.^9^, ^10^ However, the fact that these biomarkers are still experimental in combination with the high cost, limited availability, and complexity precludes their implementation in clinical practice. Hence, there is still an unmet need to establish predictive factors for development of IRAEs based on parameters easily available in daily practice.

Given the advantages of using well‐established tools in clinical practice, both performance status (PS) and Charlson comorbidity index (CCI) have been investigated as potential predictive factors for the development of IRAEs with conflicting results.^11^, ^12^, ^13^, ^14^ Age has also been investigated as a potential predictive factor for IRAEs where older patients were found to be at higher risk of experiencing IRAEs in one study^15^ but not in others.^14^, ^16^, ^17^

The simplified frailty score is an easy‐to‐use tool primarily developed from a cohort of transplant‐ineligible patients with newly diagnosed multiple myeloma to reflect patients' frailty and is based on three variables including PS, CCI, and age. Its prognostic significance has been validated^18^, ^19^, ^20^ whereas a potential predictive role for risk of chemotherapy induced severe adverse events has also been suggested.^18^ Given the simplicity of this tool, it could be easily applied in clinical practice.

The aim of the present study was to investigate whether the simplified frailty score could serve as a predictive tool for IRAEs in a cohort of patients with advanced cancer treated with CPIs.

## 2 PATIENTS AND METHODS

### 2.1 Study design and setting

In this multicenter retrospective cohort study we identified all patients treated with CPIs (PD‐1 or PD‐L1 inhibitors) for advanced cancer between January 1st 2017 until December 31st 2021 from three regions (Sörmland county, Uppsala county, and Örebro county) in Sweden. Patients treated with a combination of PD‐1 and anti‐CTLA4 inhibitor were included as well as patients treated with CPIs as part of a clinical trial.

We excluded patients treated with CPIs in a curative setting in order to make the study population more homogenous.

### 2.2 Data collection

Data were extracted from electronic medical records (EMR) by dedicated researchers in a database with pre‐specified variables of interest. The following data were collected: age at diagnosis, gender, comorbidities expressed as CCI, type of cancer, primary treatment at diagnosis, age at diagnosis of advanced cancer, metastatic sites, CPI initiation date, PS (WHO classification) at CPI initiation, exposure to antibiotics within 30 days before or after CPI initiation, exposure to steroids (> 10 mg prednisolone or equivalent dose) at the CPI initiation date, number of previous lines of treatment, best treatment response on CPI, date of disease progression, IRAEs (date, type, grade, outcome), date of death, and cause of death.

### 2.3 Outcomes and definitions

The simplified frailty score was calculated for each patient based on age, PS, and CCI (Table 1). CCI is based on following comorbidities: myocardial infarction, congestive heart failure, peripheral vascular disease, cerebrovascular accident or TIA, dementia, COPD, connective tissue disease, peptic ulcer disease, liver disease, DM, hemiplegia, chronic kidney disease, leukemia, lymphoma, AIDS, and solid tumor. For each parameter a score was set (0–2). Regarding age the score was 0 for patients ≤75 years, 1 for 76–80 years, and 2 for >80 years. PS 0 gave a score of 0, PS 1 a score of 1, and PS ≥2 a score of 2. CCI ≤1 gave a score of 0 and CCI >1 a score of 1. According to the sum of scores patients with a score 0–1 was considered nonfrail and ≥2 frail.

**TABLE 1:** Parameters in simplified frailty score.

Parameter	Score
Age	
≤75 years	0
76–80 years	1
>80 years	2
Charlson comorbidity index	
≤1	0
>1	1
ECOG performance status	
0	0
1	1
≥ 2	2
Sum of scores	
Nonfrail	0–1
Frail	≥ 2

Abbreviation: ECOG, Eastern Cooperative Oncology Group.

IRAEs were categorized in grade according to CTCAE 5.0 grading system. If the grade was not included in the EMRs, an approximation of the grade was decided based on the description of adverse events in EMRs and the laboratory findings, whenever feasible.

Multiple IRAEs were defined as IRAEs involving more than one organ system either simultaneously or sequentially.

### 2.4 Statistical methods

Categorical variables were expressed as count and frequency whereas continuous variables as median and range.

The association between potential variables of interest and risk for IRAEs (different grading levels, presence of multiple IRAEs) or discontinuation due to IRAEs was analyzed by simple and multiple logistic regression analyses to calculate odds ratios (ORs) and 95% confidence intervals (CI). Age was included as continuous variable in all analyses to avoid any arbitrary age cutoff that might impact the results. Performance status and CCI were analyzed in groups reflecting the scoring of the simplified frailty score. Variables included in the multivariate models were chosen as previously documented predictors of IRAEs and if there was a statistically significant association between the variable of interest and IRAEs in bivariate analyses.

All *p*‐values were two‐sided and a *p*‐value of <0.05 was considered statistically significant. All analyses were performed using SPSS (version 28.0‐, Released 2021. IBM SPSS Statistics for Windows, Version 28.0. IBM Corp).

## 3 RESULTS

### 3.1 Characteristics of study cohort

Baseline characteristics of the study cohort are summarized in Table 2. In total, 596 patients were included in the study cohort with a median age of 66 years (range 24–87). The most common underlying malignant disease was non‐small cell lung cancer (203 patients; 34.1%) followed by malignant melanoma (195 patients; 32.7%) and renal cell carcinoma (87 patients; 14.6%). Monotherapy with nivolumab was the most used CPI treatment (283 patients; 47.5%) followed by single treatment with pembrolizumab (209 patients; 35.1%) whereas 40 patients (6.7%) were treated with the combination of nivolumab and ipilimumab. By implementing the simplified frailty score in the study cohort, 361 patients (60.6%) were classified as non‐frail and 235 (39.4%) as frail.

**TABLE 2:** Characteristics of study cohort.

Variable	*N* (%)
Age, median (range), in years	66 (24–87)
Gender	
Male	346 (58.1)
Female	250 (41.9)
Charlson comorbidity index, median (range)	3 (0–11)
Type of cancer	
NSCLC	203 (34.1)
Melanoma	195 (32.7)
Renal cell carcinoma	87 (14.6)
Urothelial carcinoma	35 (5.9)
HNSCC	23 (3.9)
Other	53 (8.9)
Metastatic disease at diagnosis	278 (46.7)
Visceral metastases	400 (67.1)
Central nervous system metastases	50 (8.4)
Performance status according to ECOG at baseline	
0	213 (35.7)
1	278 (46.6)
2	94 (15.8)
3 or 4	11 (1.8)
Exposure to antibiotics within 30 days	118 (19.7)
Exposure to steroids at treatment initiation	47 (7.8)
Type of checkpoint inhibitors	
Nivolumab	283 (47.5)
Pembrolizumab	209 (35.1)
Atezolizumab	56 (9.4)
Nivolumab + ipilimumab	40 (6.7)
Durvalumab	4 (0.7)
Cemiplimab	4 (0.7)
Line of treatment for checkpoint inhibitors	
First	268 (45.0)
Second	231 (38.8)
Third or later	97 (16.3)
Simplified frailty score	
Nonfrail	361 (60.6)
Frail	235 (39.4)

Abbreviations: ECOG, Eastern Cooperative Oncology Group; HNSCC, head and neck squamous cell carcinoma; NSCLC, non‐small cell lung cancer.

### 3.2 Frequency and outcome of IRAEs

Table 3 presents the frequency of IRAEs in frail and non‐frail patients according to the simplified frailty score. Any grade IRAEs, grade ≥2 IRAEs and multiple IRAEs were significantly more common in the frail patient group with univariate ORs of 1.93 (95% CI: 1.38–2.70), 1.60 (95% CI: 1.14–2.46), and 2.05 (95% CI: 1.32–3.17), respectively.

**TABLE 3:** Frequency of immune‐related adverse events using simplified frailty score.

	Nonfrail (%) *N* = 361	Frail (%) *N* = 235
Any grade IRAE	155 (42.9)	138 (58.7)
IRAE grade ≥2	117 (32.4)	102 (43.4)
IRAE grade ≥3	58 (16.1)	46 (19.6)
Multiple IRAEs	45 (12.5)	53 (22.6)
Discontinuation due to IRAE	60 (16.6)	43 (18.3)

Abbreviation: IRAE, immune‐related adverse events.

**FIGURE 1:**
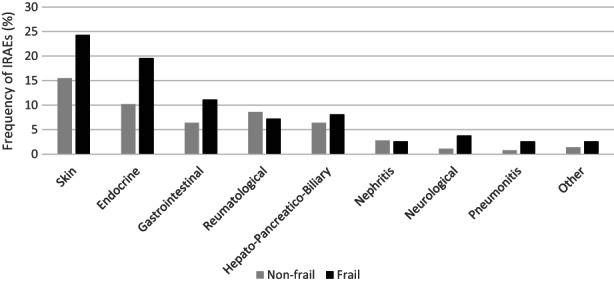
Frequency of any grade immune‐related adverse events (IRAEs) per frailty status according to simplified frailty score.

The distribution of IRAEs by target organ in frail and non‐frail patient cohorts was similar with skin IRAEs being most frequent in both cohorts followed by endocrine‐related IRAEs while gastrointestinal, rheumatological, and hepatopancreaticobiliary IRAEs were within top 3–5 in both frail and non‐frail patients (Figure 1).

Regarding the outcome of IRAEs, no numerical differences in the rates of resolution or worsening between the two patient groups were observed (Table 4). Four nonfrail (2.9%) and one frail (0.6%) patient died due to IRAE (*p*‐value = 0.653). Three of these patients were treated with a combination of nivolumab and ipilimumab and two with monotherapy. Three of the patients had an underlying hepatopancreaticobiliary IRAE with lethal outcome whereas one patient had a gastrointestinal IRAE, and one had a neurological IRAE.

**TABLE 4:** Outcome of IRAEs in relation to simplified frailty score.

Outcome of IRAEs	Non‐frail with any IRAE (%) *N* = 138	Frail with any IRAE (%) *N* = 155
Resolved without sequelae	78 (56.5)	86 (55.5)
Resolved with minor sequelae	47 (34.1)	55 (35.5)
Resolved with major sequelae	7 (5.1)	7 (4.5)
Worsening	2 (1.4)	6 (3.9)
Death due to IRAE	4 (2.9)	1 (0.6)

Abbreviation: IRAEs, immune‐related adverse events.

### 3.3 Predicting IRAEs

We investigated the potential predictive role of age, CCI, and PS as independent factors but also combined as simplified frailty score on the risk for IRAEs or treatment discontinuation due to IRAEs. The results of simple and multiple regression analyses are summarized in Table 5.

**TABLE 5:** Impact of age, performance status, and simplified frailty score on predicting immune‐related toxicities.

Outcome	Age (continuous)		Performance status (0 vs. 1 vs. ≥2)		Charlson comorbidity index (0–1 vs. 2–3 vs. >3)		Simplified frailty score (nonfrail vs. frail)^d^	
	Univariate OR (95% CI)	Multivariate^a^ OR (95% CI)	Univariate OR (95% CI)	Multivariate^b^ OR (95% CI)	Univariate OR (95% CI)	Multivariate^c^ OR (95% CI)	Univariate OR (95% CI)	Multivariate OR (95% CI)
Any grade IRAE	0.96 (0.98–1.01)	1.00 (0.98–1.03)	1.13 (0.57–2.93) 1.92 (0.96–4.66)	1.18 (0.82–1.72) 1.85 (0.98–3.02)	0.77 (0.53–1.12) 1.14 (0.73–1.79)	0.86 (0.46–1.62) 1.27 (0.60–2.70)	**1.93 (1.38–2.70)**	**1.58 (1.09–2.28)**
IRAE grade ≥2	0.99 (0.98–1.01)	1.00 (0.98–1.03)	1.41 (0.85–2.33) 2.11 (0.96–3.68)	1.44 (0.86–2.42) 1.95 (0.93–3.49)	0.71 (0.48–1.05) 1.25 (0.80–1.96)	0.67 (0.36–1.26) 1.06 (0.50–2‐25)	**1.60 (1.14–2.46)**	1.30 (0.89–1.90)
IRAE grade ≥3	1.00 (0.98–1.02)	1.01 (0.98–1.04)	1.69 (0.82–3.47) 1.37 (0.71–2.65)	1.46 (0.69–3.09) 1.32 (0.67–2.61)	0.56 (0.36–1.01) 1.02 (0.59–1.75)	0.60 (0.27–1.32) 1.13 (0.46–2.87)	1.27 (0.83–1.95)	1.00 (0.60–1.57)
Multiple IRAEs	0.99 (0.97–1.00)	1.01 (0.98–1.04	1.80 (0.81–4.02) **2.76 (1.19–6.39)**	1.77 (0.79–4.01) 2.42 (0.94–5.74)	0.75 (0.44–1.27) **1.78 (1.03–3.08)**	0.68 (0.38–1.21) 1.44 (0.57–3.64)	**2.05 (1.32–3.17)**	**1.62 (1.00–2.64)**
Discontinuation due to IRAE	1.02 (1.00–1.04)	1.02 (0.99–1.05)	1.09 (0.57–2.01) 2.19 (0.98–4.36)	1.06 (0.54–2.07) 1.93 (0.95–3.93)	0.70 (0.38–1.30) 1.05 (0.66–1.71)	1.45 (0.63–3.35) 1.28 (0.47–3.49)	1.12 (0.73–1.73)	0.92 (0.57–1.49)

*Note:* Statistically significant associations are highlighted with bold.

Abbreviation: IRAEs, immune‐related adverse events.

^a^ Adjusted for gender, Charlson comorbidity index, performance status, cancer type, type of checkpoint inhibitors, line of treatment.

^b^ Adjusted for gender, age, Charlson comorbidity index, cancer type, type of checkpoint inhibitors, line of treatment.

^c^ Adjusted for gender, age, performance status, cancer type, type of checkpoint inhibitors, line of treatment.

^d^ Adjusted for gender, cancer type, type of checkpoint inhibitors, line of treatment.

Neither age, nor CCI or PS were independently predictive of IRAEs or treatment discontinuation due to IRAEs in multivariate analyses.

In terms of simplified frailty score, frail patients were at a significantly higher risk for developing an IRAE irrespective of grade (OR: 1.58; 95% CI: 1.09–2.28) and multiple IRAEs (OR: 1.62; 95% CI: 1.00–2.64) but not for high‐grade IRAEs or treatment discontinuation.

## 4 DISCUSSION

The aim of this retrospective multicenter cohort study was to investigate whether a simplified frailty score, based on three variables that are easily available in clinical practice (age, PS, and CCI), could serve as a predictive tool for IRAEs in patients with advanced cancer receiving CPIs. To our knowledge this is the first study to evaluate the correlation between IRAEs and the simplified frailty score earlier used in myeloma patients to predict clinical outcomes.^18^ The current study describes data from a real‐world population of almost 600 patients and shows that IRAEs regardless of grade and multiple IRAEs are significantly more common in frail patients according to the simplified frailty score whereas age, CCI, or PS did not separately predict the development of IRAEs.

Considering that age, the presence of comorbidities, and PS are well‐established factors associated with lower tolerance for chemotherapy^21^, ^22^, ^23^ these factors have also been studied separately in cancer patients receiving CPIs.^12^, ^14^, ^15^, ^16^, ^17^, ^24^, ^25^

Although age has been investigated as a potential factor associated with increased risk for IRAEs^15^ such an association has not been observed in pivotal randomized trials^25^ nor in real‐world evidence studies.^14^, ^16^, ^17^, ^24^ In line with these studies, age alone was not associated with development of IRAEs or discontinuation of therapy in our study.

Although patients with different comorbidities including autoimmune diseases were excluded from initial clinical trials, real‐world data suggest that CPIs may be feasible in patients with low‐risk autoimmune disease such as rheumatoid arthritis and psoriasis, mild to moderate hepatic and renal dysfunction, and certain chronic viral infections.^12^, ^26^, ^27^, ^28^ The results from these studies correspond to our observation that comorbidity, measured as CCI, did not by itself predict IRAEs.

The Eastern Cooperative Oncology Group (ECOG) PS is routinely used to assess tolerance to chemotherapy and to predict survival.^22^, ^29^, ^30^ Patients with ECOG PS >1 are frequently excluded from pivotal randomized trials and outcomes in this group are therefore mainly based on retrospective studies from real world settings. Treatment with CPIs seems to be safe and effective also in patients with poor PS even though response rates and overall survival in this group is clearly inferior compared to patients with PS 0–1.^16^, ^31^, ^32^ This observation is supported by our results indicating that PS as a single variable does not predict the development of IRAEs.

There are a few previous studies investigating the correlation between frailty and the development of IRAEs. One of these studies assessed the safety and tolerability of CPIs in older cancer patients divided into frailty groups according to a non‐validated frailty tool based on PS, CCI, and neutrophil‐lymphocyte ratio and found no increase of IRAEs among patients in the high‐frailty group.^33^ Geriatric assessment through validated questionnaires as the Geriatric 8 (G8), containing eight questions, can be used to predict toxicity of treatment with chemotherapy, radiation therapy, and endocrine therapy.^34^, ^35^, ^36^, ^37^ Bruijnen et al. conducted a study among stage III and IV melanoma patients aged 70 years and older treated with anti‐PD‐1 monotherapy, where G8 did not predict IRAEs.^38^ Similarly, the study of Welaya et al. failed to demonstrate any association between impairment in any geriatric assessment domain and IRAEs in a pilot study including older cancer patients treated with CPIs.^39^

The current study has several limitations. First, the retrospective nature of the study makes the results more susceptible to bias. As a consequence of the study's retrospective design, the identification of IRAEs was based on information in EMRs and there is a risk that low‐grade IRAEs are underreported. Meanwhile, the frequencies of both any‐grade and grade ≥3 IRAEs, with only a small minority having received combination immunotherapy (6.7%) and the lethal outcome of immune‐related toxicity (0.8%) in our cohort is similar as reported in randomized trials.^40^ Another potential source of bias is the risk for misclassification regarding grade of IRAE as well as the risk for a single IRAE with consequences being classified as multiple IRAEs. Furthermore, the limited number of events precludes any reliable analyses in terms of the potential role of frailty score in predicting site‐specific IRAEs or grade 5 IRAEs. Finally, the simplified frailty score was developed for myeloma patients and treatments for myeloma differ a lot from immunotherapy, therefore the score might not reflect aspects of importance for patients treated with CPIs.

In summary, the current study shows that IRAEs regardless of grade and multiple IRAEs are significantly more common in frail patients according to the simplified frailty score. At the same time, the three factors constituting the frailty score, that is, age, CCI, or PS did not independently predict development of IRAEs. However, the difference in IRAEs between frail and nonfrail patients did not reach statistical significance for patients experiencing severe (grade 3–4) toxicity or treatment discontinuation constituting the most clinically relevant outcomes. Considering the lack of easy‐to‐use predictive tools for IRAEs in clinical practice and our preliminary findings, the simplified frailty score may be of value in clinical decision making for patients who are eligible for CPIs and deserves further validation through a large prospective study.

## AUTHOR CONTRIBUTIONS

**Cecilia Olsson Ladjevardi:** Data curation (equal); writing – original draft (lead). **Anthoula Koliadi:** Data curation (equal); writing – review and editing (supporting). **Viktoria Rydén:** Data curation (equal); writing – review and editing (supporting). **Ali Inan El‐Naggar:** Data curation (equal); writing – review and editing (supporting). **Evangelis Digkas:** Data curation (equal); writing – review and editing (supporting). **Antonis Valachis:** Conceptualization (lead); data curation (lead); formal analysis (lead); funding acquisition (equal); methodology (lead); project administration (lead); software (lead); supervision (lead); validation (lead); writing – original draft (lead). **Gustav Ullenhag:** Conceptualization (supporting); data curation (supporting); formal analysis (supporting); funding acquisition (lead); methodology (supporting); project administration (equal); supervision (lead); validation (supporting); writing – original draft (lead).

## FUNDING INFORMATION

The study was supported by grants to Prof. G.J. Ullenhag from The Research Foundation Stiftelsen Onklologoska Kliniken i Uppsalas Forskningsfond and Uppsala University Hospital (ALF).

## CONFLICT OF INTEREST STATEMENT

None of the authors has any potential financial conflict of interest related to this manuscript.

## ETHICS STATEMENT

The study has been approved from the Swedish Ethical Review Authority (2019‐02469). Considering the retrospective nature of the study, the Ethical Review Authority waived the need for informed consent.

## Data Availability

Data available on request due to privacy/ethical restrictions.
